# Supplementary material for: Hybrid Models and Biological Model Reduction with PyDSTool
Source: PLoS Comput Biol. 2012 Aug 9;8(8):e1002628. doi: 10.1371/journal.pcbi.1002628 (PMC3415397; doi:10.1371/journal.pcbi.1002628)
Supplement: Text S4 — Complete source code for the PyDSTool package (version 0.88.120504). Includes API documentation and help files linking to web pages. This file is identical to the current public release on Sourceforge.net. (ZIP) [file pcbi.1002628.s004.zip › PyDSTool/html/PyDSTool.Toolbox.synthetic_data-pysrc.html]

xml version="1.0" encoding="ascii"?


PyDSTool.Toolbox.synthetic\_data


| Home | Trees | Indices | Help | PyDSTool |
| --- | --- | --- | --- | --- |

Package PyDSTool ::
Package Toolbox ::
Module synthetic\_data

[hide private]

[frames] | no frames]

# Source Code for Module PyDSTool.Toolbox.synthetic\_data

```
  1  """Helper functions for creating synthetic data 
  2   
  3  Robert Clewley, 2006, 2007. 
  4   
  5  """ 
  6   
  7  from PyDSTool import * 
  8  from numpy import random, array, dot, zeros, transpose 
  9  from numpy.linalg import norm, inv 
 10   
 11  _generators = ['generate_swirl', 'generate_discspiral', 'generate_hypercube', 
 12                'generate_spiral'] 
 13   
 14  _hypercuboid_utils = ['sup_norm', 'inf_norm', 'maxs', 'mins', 
 15                        'max_euclidean_distance'] 
 16   
 17  __all__ = _generators + _hypercuboid_utils 
 18   
 19  # ---------------------------------------------------------------------------- 
 20   


21 -def generate_swirl(N, L, zmin, zmax, cycle_rate, bias_x, bias_y, roll_num=0, 
 22                     noise=0, fromcentre=True):


23      """Generate N points on a 3D 'swirl' (a cyclic trajectory on a 
 24      1 x L "swiss roll" manifold). 
 25   
 26      cycle_rate is measured in radians. 
 27      roll_num is the number of full rolls in the swiss roll. 
 28   
 29      The origin is at the centre of the set unless fromcentre=False. 
 30      """ 
 31      X = zeros((N, 3), float) 
 32      r = 0.5 
 33      assert L > 0 
 34      assert zmax >= 1 
 35      assert zmin > 0 
 36      assert zmax > zmin 
 37      zscale = (zmax - zmin)/L 
 38      def roll(x): 
 39          r = zmax - x*zscale 
 40          rho = x/L*(roll_num*2*pi) 
 41          return (r*cos(rho), r*sin(rho))

 42      theta = 0 
 43      for i in xrange(N): 
 44          theta += cycle_rate 
 45          x = r*cos(theta)+bias_x*i 
 46          x_rolled, z_rolled = roll(x) 
 47          X[i,:] = array([x_rolled + random.normal(0,noise), 
 48                          r*sin(theta) + bias_y*i + random.normal(0,noise), 
 49                          z_rolled + random.normal(0,noise)]) 
 50      return X 
 51   
 52   


53 -def generate_discspiral(N1, N2, D, radius, cycle_rate, 
 54                          num_spirals=1, noise=0):


55      """Generate N1 points on a planar disc and N2 points on up to two spirals 
 56      attached to it, also in the plane. The data are embedded in an ambient 
 57      space of dimension D. 
 58      The cycle_rate (radians per point) determines how much of the spiral is 
 59      created. 
 60      num_spirals can be 0, 1, or 2 
 61      """ 
 62      assert D > 1 
 63      assert num_spirals in [0,1,2] 
 64      X = zeros((N1+num_spirals*N2, D), float) 
 65      # first take care of the disc 
 66      assert radius >= 1 
 67      X[0:N1,:2] = generate_ball(N1, 2, radius) 
 68      for i in range(N1): 
 69          X[i,2:D] = array([random.normal(0,noise)]*(D-2), float) 
 70      # now do the spiral(s) 
 71      theta = sqrt(radius*radius-1) 
 72      for i in xrange(N2): 
 73          theta += cycle_rate 
 74          r = sqrt(theta*theta+0.8) 
 75          X[N1+i,:] = array([r*cos(theta)+random.normal(0,noise), 
 76                             r*sin(theta)+random.normal(0,noise)] + \ 
 77                            [random.normal(0,noise)]*(D-2)) 
 78          if num_spirals == 2: 
 79              X[N1+N2+i,:] = array([r*cos(theta+pi)+random.normal(0,noise), 
 80                                 r*sin(theta+pi)+random.normal(0,noise)] + \ 
 81                                   [random.normal(0,noise)]*(D-2)) 
 82      return X

 83   
 84   


85 -def generate_spiral(N, D, radius, cycle_rate, expand_rate=1., 
 86                          num_spirals=1, noise=0):


87      """Generate N points on up to two spirals in the plane. 
 88      The data are embedded in an ambient space of dimension D. 
 89      The cycle_rate (radians per point) determines how much of the spiral is 
 90      created. 
 91      radius is the starting radius of the spiral. 
 92      num_spirals can be 0, 1, or 2 
 93      """ 
 94      assert D > 1 
 95      assert num_spirals in [0,1,2] 
 96      X = zeros((num_spirals*N, D), float) 
 97      theta = sqrt(radius*radius-1) 
 98      for i in xrange(N): 
 99          theta += cycle_rate 
100          r = sqrt(theta*theta+0.8)*expand_rate 
101          X[i,:] = array([r*cos(theta)+random.normal(0,noise), 
102                             r*sin(theta)+random.normal(0,noise)] + \ 
103                            [random.normal(0,noise)]*(D-2)) 
104          if num_spirals == 2: 
105              X[N+i,:] = array([r*cos(theta+pi)+random.normal(0,noise), 
106                                 r*sin(theta+pi)+random.normal(0,noise)] + \ 
107                                   [random.normal(0,noise)]*(D-2)) 
108      return X

109   


110 -def generate_hypercube(N, D, length, fromcentre=True):


111      """Generate a length-N uniformly-distributed random set of data points in a 
112      D-dimensional hypercube having side-length given by third parameter. 
113   
114      The origin is at the centre of the set unless fromcentre=False. 
115      """ 
116      X = zeros((N, D), float) 
117      if fromcentre: 
118          for i in xrange(N): 
119              X[i,:] = array([random.uniform(-length/2., length/2.) for j in xrange(D)], 
120                             float) 
121      else: 
122          for i in xrange(N): 
123              X[i,:] = array([random.uniform(0, length) for j in xrange(D)], 
124                             float) 
125      return X

126   


127 -def generate_ball(N, D, radius):


128      """Generate a length-N uniformly-distributed random set of data points in a 
129      D-dimensional ball of radius given by the third parameter. Points are found 
130      by elimination of points from hypercubes and thus this method is not 
131      recommended for large D! 
132      """ 
133      X = zeros((N, D), float) 
134      twopi = 2*pi 
135      assert D > 1 
136      foundpts = 0 
137      outsidepts = 0 
138      while foundpts < N: 
139          hcube = generate_hypercube(N, D, 2*radius) 
140          for x in hcube: 
141  ##            xr = norm(x) 
142              if norm(x) < radius: 
143                  X[foundpts,:] = x 
144                  foundpts += 1 
145  ##                print x, xr, "inside" 
146                  if foundpts == N: 
147                      break 
148              else: 
149                  outsidepts += 1 
150  ##                print x, xr, "outside" 
151      print "Number of points outside ball that were discarded = ", outsidepts 
152      return X

153   
154   
155  # ----------------------------------------------------------------------------- 
156   
157  # Utilities for metrics on synthetic data distributed in a hypercuboid 
158   


159 -def sup_norm(p, lengths=None):


160      """sup norm for data distributed in a hypercuboid with given lengths.""" 
161      return max(maxs(p), lengths)

162   


163 -def inf_norm(p, lengths=None):


164      """inf norm for data distributed in a hypercuboid with given lengths.""" 
165      return min(mins(p), lengths)

166   


167 -def maxs(p, lengths=None):


168      """Return ordered array of max distances from a point to the edges of 
169      a hypercuboid. 
170      """ 
171      if lengths is None: 
172          lengths = [1.] * len(p) 
173      a = array([max([p[i],lengths[i]-p[i]]) for i in xrange(len(p))]) 
174      a.sort() 
175      return a

176   


177 -def mins(p, lengths=None):


178      """Return ordered array of min distances from a point to the edges of 
179      a hypercuboid. 
180      """ 
181      if lengths is None: 
182          lengths = [1.] * len(p) 
183      a = array([min([p[i],lengths[i]-p[i]]) for i in xrange(len(p))]) 
184      a.sort() 
185      return a

186   


187 -def max_euclidean_distance(lengths):


188      """Returns the largest Euclidean distance possible in a D-dimensional 
189      hypercuboid with given side-lengths.""" 
190      return norm(lengths, 2)

191
```

  


| Home | Trees | Indices | Help | PyDSTool |
| --- | --- | --- | --- | --- |

|  |  |
| --- | --- |
| Generated by Epydoc 3.0.1 on Fri May 4 15:24:22 2012 | http://epydoc.sourceforge.net |
